# Vaccarin hastens wound healing by promoting angiogenesis via activation of MAPK/ERK and PI3K/AKT signaling pathways *in vivo* ^1^

**DOI:** 10.1590/s0102-865020190120000002

**Published:** 2020-02-07

**Authors:** Bao Hou, Weiwei Cai, Ting Chen, Zhixuan Zhang, Haifeng Gong, Wei Yang, Liying Qiu

**Affiliations:** I Assistant Experimentalist, Department of Basic Medicine, Wuxi School of Medicine, Jiangnan University, Wuxi, China. Technical procedures, manuscript preparation.; II Experimentalist, Department of Basic Medicine, Wuxi School of Medicine, Jiangnan University, Wuxi, China. Histological examinations, manuscript preparation.; III Master, Department of Basic Medicine, Wuxi School of Medicine, Jiangnan University, Wuxi, China. Acquisition of data, statistics analysis.; IV PhD, Professor, Department of Basic Medicine, Wuxi School of Medicine, Jiangnan University, Wuxi, China. Critical revision, final approval.

**Keywords:** Vaccaria, Angiogenesis Inducing Agents, Wound Healing, Rats

## Abstract

**Purpose:**

To explore the potential role and unclear molecular mechanisms of vaccarin in wound healing.

**Methods:**

Rats’ skin excision model to study the effects of vaccarin on wound healing *in vivo* . Hematoxylin and eosin staining was performed to evaluate Histopathologic characteristics. Immunohistochemistry was employed to assess the effects of vaccarin in accelerating angiogenesis. Western blot was used to evaluate relative protein expressed levels.

**Results:**

Vaccarin could significantly promote wound healing and endothelial cells and fibroblasts proliferation in the wound site. Immunohistochemistry and Western blot studies showed that the nodal proteins and receptor (bFGFR) related to angiogenesis signaling pathway were activated, and the microvascular density in the wound site was markedly higher than that in the control group.

**Conclusions:**

The present study was the first to demonstrate that vaccarin is able to induce angiogenesis and accelerate wound healing *in vivo* by increasing expressions of p-Akt, p-Erk and p-bFGFR. This process is mediated by MAPK/ERK and PI3K/AKT signaling pathways.

## Introduction

The skin is the largest organ in the human body, accounting for 15% of body weight approximately^1 , 2^ {Mulholland, 2017 #122;Tobin, 2006 #191;Tobin, 2006 #225}. It plays an important role in protecting our body against the external environment. Convincing evidence showed that wound healing is still a problem that needs to be solved urgently, especially the chronic ulcers and diabetic wounds healing^3^ . A 2009 study had demonstrated that nearly 6.5 million patients suffer from the disturb of chronic wounds every year^4^ . Wound healing is not only a normal physical response, but also a multifaceted process involving orchestrated cellular signaling events and biochemical cascades^5 , 6^ . Wound healing mainly consists of four overlapped phases, including homeostasis, inflammation, proliferation and reorganization^6^ . Any defects at these stages will contribute to a negative impact on the healing process, resulting in wound healing becoming a major social problem. Although some research has been carried out on wound healing, no better means have been found to treat it.

FGFRs are receptor tyrosine kinases that play a critical role in several biological processes, such as metabolic homeostasis, tissue repair and regeneration^7^ . In normal cells, the FGFR signaling pathway is involved in key cell behaviors, including proliferation, differentiation, migration, and survival, and is a pivotal regulator of angiogenesis and wound healing in adults^8 - 10^ . However, FGFR1 is mainly expressed in endothelial cells^10^ . FGF-2 is one of 23 structurally related polypeptide growth factors^11^ . FGF-2, as a basic FGF pI (>9.0) , is a response for modulating numerous cellular functions in multiple cell types, including cell proliferation, differentiation and blood vessel remodeling^12^ . Therefore, activated FGF-2/FGFR-1 signaling may open up a novel strategy for neovascular disease therapeutics.

**Figure 1:**
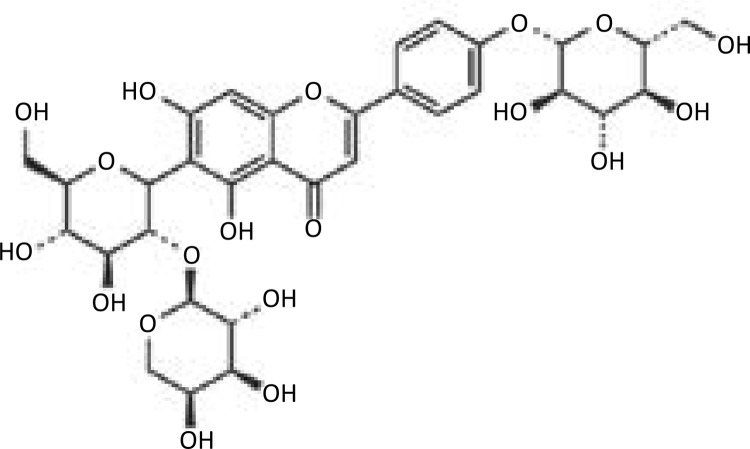
Chemical structure of vaccarin.

Traditional Chinese medicine has a variety of targets and various forms of intervention for wound healing characteristics^13^ . Vaccarin is the main active component of *Vaccaria segetalis (Neck.)* Garcke. ex Asch. (Caryophyllaceae). Vaccarin is a single compound extracted from Vaccaria segetalis seeds ( Fig. 1 ). Recently, in vitro studies have shown that vaccarin can stimulate endothelial cell proliferation and migration and tube formation of HMEC-1^14^ . It has also been found that vaccarin has an angiogenic effect by constructing a matrigel plug model in mice^15^ . In addition, Sun *et al* .^16^ report that vaccarin promotes angiogenesis by activating the fgf-2-mediated FGFR1 signaling pathway in human microvascular endothelial cells. The existing evidence highlights that neovasculogenesis is crucial for wound healing, because it involves the delivery of oxygen, nutrients, and other mediators at the wound site^17^ . All the above studies hinted that vaccarin may display positive effects on wound healing. However, knowledge with regard to the effects of vaccarin on wound healing is still unclear. Therefore, we aimed to explore the potential role and underlying molecular mechanisms of vaccarin in wound healing.

## Methods

### Chemicals

Vaccarin was obtained from Shanghai Shifeng Technology Co., Ltd. (Shanghai, China). The CD31 (AB60701a) rabbit polyclonal antibody was purchased from Sangon Biotec (Shanghai) Co, Ltd.(Shanghai, China). p-VEGFR was bought from Affinity (cat.no.AF3280). PCNA was purchased from Wanleibio (cat.no.WL0341c) p-Erk (ab76165) and p-Akt (ab66138) rabbit polyclonal antibodies were purchased from Abcam. p-bFGFR was purchased from Santa Cruz Biotechnology, Inc. Goat anti-rabbit secondary immunoglobulin (Ig)G antibody was obtained from Sangon Biotech Co., Ltd. The SABC kit was obtained from Nanjing Jiancheng Biological Technology, Inc. Hematoxylin and eosin were purchased from Wuhan booster biological engineering technology Co., LTD. MEBO was purchased from Shantou MEBO pharmaceutical Co., LTD.

### Animals

Eight-week-old SD (Sprague-Dawley) male rats (200-220 g) were obtained from the Research Center of Laboratory Animals (Hangzhou, China; grade specific-pathogen free and certificate no. SCXK (Zhe) 2008-0033). The rats were maintained in a barrier facility, in a temperature-and humidity-controlled environment, fed sterilized food and were given ad libitum access to water. Prior to the experiment, all of the rats were allowed to acclimate for one week. All of the procedures and protocols conformed to Good Publishing Practice in planta medica .The study was approved by the Animal Care Committee of Jiangnan University. Approval number: JN.No20180330i0520601.

### Animal model and surgical procedure

16 SD male rats aged 8 weeks were used in this study and divided into two groups: the vehicle control and vaccarin treated group. The animals were anesthetized and then hair on the back was clipped, and the skin was washed with povidone solution and wiped with sterile water. The 1cm diameter full-thickness skin excision wounds were made on both sides in the rats’ back. 0.02 g of vehicle ointments was added to the left wound site and ointments with 0.1% vaccarin were added to the right wound site. We measured and recorded wound areas every day. On the 3th, 6th, 9th and 11th day, we killed four animals in each group, then took traumatic skin fixed in 4% buffered paraformaldehyde.

Woundclosurepercentage(%)=[(areaonday0−openareaondayn)/areaonday0]×100.

MEBO (Moist Exposed Burn Ointment (MEBO) was selected as the positive control, and the data were presented in the supplementary material. MEBO is widely used as topical agent applied on skin burn, and is a Chinese burn ointment that was first developed at the China National Science and Technology Centre in Beijing in 1989. It is a topical, easy to apply agent irrespective of site, extent, and local condition of the wound^18^ .

### Histopathologic evaluation by hematoxylin and eosin staining

The wound specimens including full thickness skin lays (epidermis, dermis, and hypodermis) were fixed in 4% buffered paraformaldehyde and processed according to the routine light microscope tissue processing methods, and the processed tissues were embedded in paraffin. Five micrometer thick skin tissue sections were stained with hematoxylin and eosin (H&E), as per standard method and visualized under light microscope (Nikon ECLIPSE 80i, Olympus, Japan) at magnification ×10 and ×40. Ten random fields (n = 10) of two stained sections of each group were evaluated from central healing areas in a blind fashion at ×40.

### Immunohistochemistry and immunofluorescence analysis

Immunohistochemistry: The skin tissue sections at the same site were placed on treated slides. According to sequences, sections were fixed, deparaffinized and rehydrated in distilled water. Tissues were boiled in 2% citrate buffer at 95˚C for 20 min, then treated with 3% hydrogen peroxide in order to block the activity of endogenous peroxidase. The slides were incubated with protein-blocking agent, then treated with the primary antibodies, including p-bFGFR (1:50), p-VEGFR (1:50), PCNA(1:100), CD31 (1:25), p-Erk (1:40) and p-Akt (1:40) at 4˚C overnight. The tissues were then incubated with the secondary biotinylated IgG antibodies (1:2,000) and finally stained with 3,3’-diaminobenzidine according to the manufacturer’s instructions. Counterstaining was performed with hematoxylin. Microvascular density (MVD) was determined, as specified by Weidner. p-bFGFR, p-VEGFR, p-Erk and p-Akt expression was determined using Image-Pro Plus 6.0 software (Media Cybernetics, Rockville, MD, USA).

Immunofluorescence: Immunofluorescence analyses of bFGFR and CD31 expression were performed with primary antibodies include anti-bFGFR (1:50) and anti-CD31 (1:25). After washing three times in TBS/T, the specimens were incubated in the dark with either FITC-conjugated anti-rabbit IgG or Cy3-conjugated anti-mouse IgG. Finally, after washing three times in TBS/T, the coverslips were mounted onto confocal microscopy with aNikon ECLIPSE 80i microscope to detect bFGFR and CD31 expression, respectively.

### Western blot analysis

Protein levels were analyzed by western blot. Briefly, 25µg total protein/well was loaded after denaturing in loading buffer at 100˚C for 5 min. The protein extracts were subjected to 8-12% SDS-PAGE and transferred to a nitrocellulose membrane (Millipore, Billerica, MA, USA). Following the transfer, the membranes were blocked at room temperature for 2 h in 5% skimmed dry milk/TBST and were incubated at 4˚C overnight with various primary antibodies. The primary antibodies are as follows: p-bFGFR (1:500 dilution), bFGFR (1:500 dilution), Akt (1:1000 dilution) and p-Akt (1:1000 dilution), Erk (1:1000 dilution) and p-Erk (1:1000 dilution), β-tublin (1:1000 dilution). The following day, the membranes were washed three times with TBST for 10 min at room temperature, and were subsequently incubated in secondary antibody (anti-rabbit immunoglobulin G, 1:2000 dilution) conjugated to horseradish peroxidase for 2 h at room temperature. Following incubation, the membranes were washed as above, and the protein bands were visualized using the DAB-advanced western blotting detection kit. β-tublin was used as the protein loading control.

### Statistical analysis

All data were expressed as mean ± standard error (SD). The significance was calculated by Student’s t-test as indicated by the *P* value. *P* values less than 0.05 ( *P* < 0.05) were considered statistically significant.

## Results

### Effects of vaccarin on wound healing in vivo

**Figure 2:**
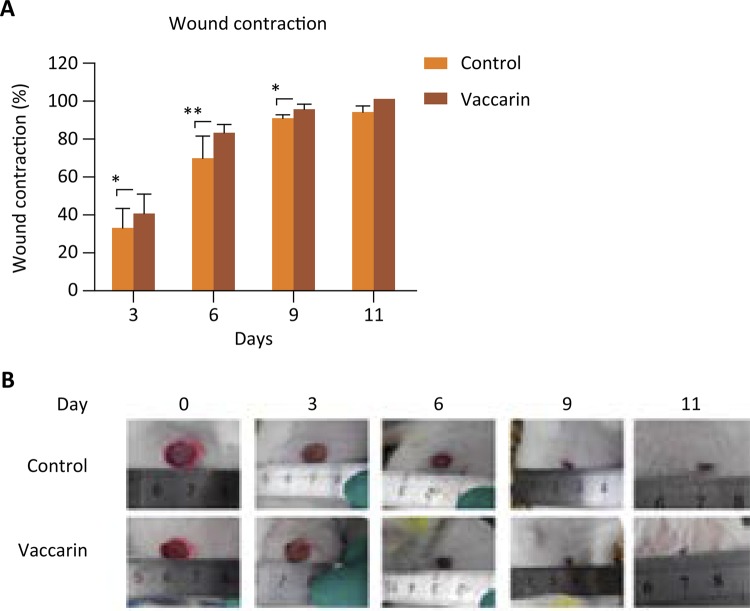
Vaccarin promoted wound healing. The 1 cm diameter full-thickness skin excision wounds were made on both sides in SD rats’ back. 0.02 g of vehicle ointments was added to the left wound site and ointments with 0.1% vaccarin were added to the right wound site. (A) The wound closure percentage (%) post wounding. Vaccarin significantly contributed to the wound healing compared with the vehicle control group. Results are expressed as mean ± SD of eight independent experiments. (* *P* < 0.05, ** *P* < 0.01, versus control). (B) The wound appearance observed at different time points.

First, we assessed the role of vaccarin on promoting wound healing in vivo. Wound healing of the skin excision was determined by the percentage of wound surface covered by regenerating epidermis. As shown in Figure 2A , vaccaricn significantly contributed to the wound healing, compared to the control group ( *P* < 0.05–0.01). The appearance of the repaired wounds sites is shown in Figure 2B . As can be seen from the figure, wounds treated by vaccarin recovered much more quickly showing better skin appearance. At the 9th day, the wounds in the vaccarin treatment group were almost recovered, but the vehicle control group was still obvious. These results indicate that vaccarin may be a promising candidate for wound healing.

### Effects of vaccarin on histopathology of wound skin

**Figure 3:**
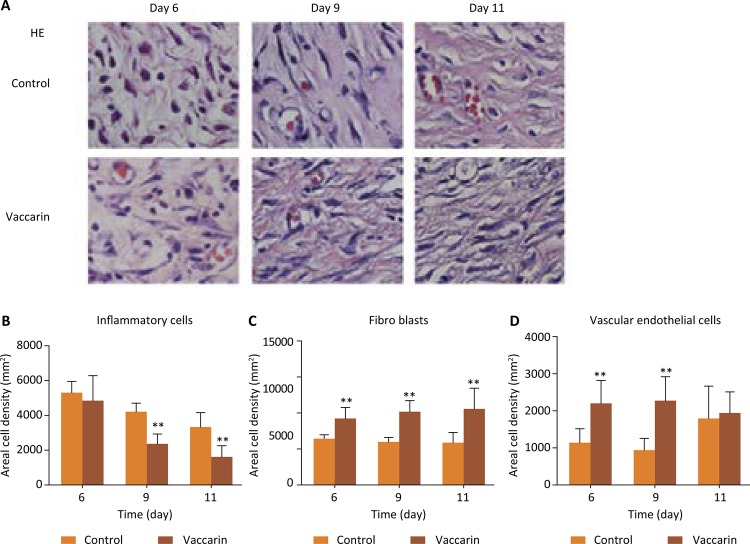
Vaccarin ameliorated the pathological tissue of wound skin. (A) Representative images of H&E-stained histologic wound sections of control and vaccarin-treated rats on the 6th, 9th and 11th day after wounding (magnification x400). (B-D) Representative the inflammatory cells, fibroblasts and endothelial cell count from 10 randomly chosen high-power fields (magnification x400) from H&E-stained cutaneous wound sections.

To further explore the role of vaccarin on histopathology of wound skin, we observed the pathological changes of the skin at the wound site by HE staining. As can be seen from the Figure 3 (A-D), on the 6th day, the vaccarin treated group exhibited uniform and thick granulation tissue formation with marked proliferation of fibroblasts, collagen deposition and few inflammatory cells. However, the wound area of the vehicle control group was dominated by inflammatory cells. On the 9th day, granulation tissue became thicker due to fibroblasts proliferation and collagen deposition in the vaccarin-treated group, while the control group exhibited some fibroblasts and few inflammatory cells. On the 11th day, the vaccarin treated group showed thick compact extracellular matrix covered by the epithelial layer resembling normal skin and some blood vessels also appeared. At the same time, the control group exhibited some collagen deposition, blood vessels and thick granulation tissue. Further analysis showed that proliferation of blood vessels and fibroblasts were more present along with adequate collagen deposition in the vaccarin treated group, which suggested that better quality granulation tissue was formed by the topical application of vaccarin.

### Effects of vaccarin on cell proliferation

**Figure 4:**
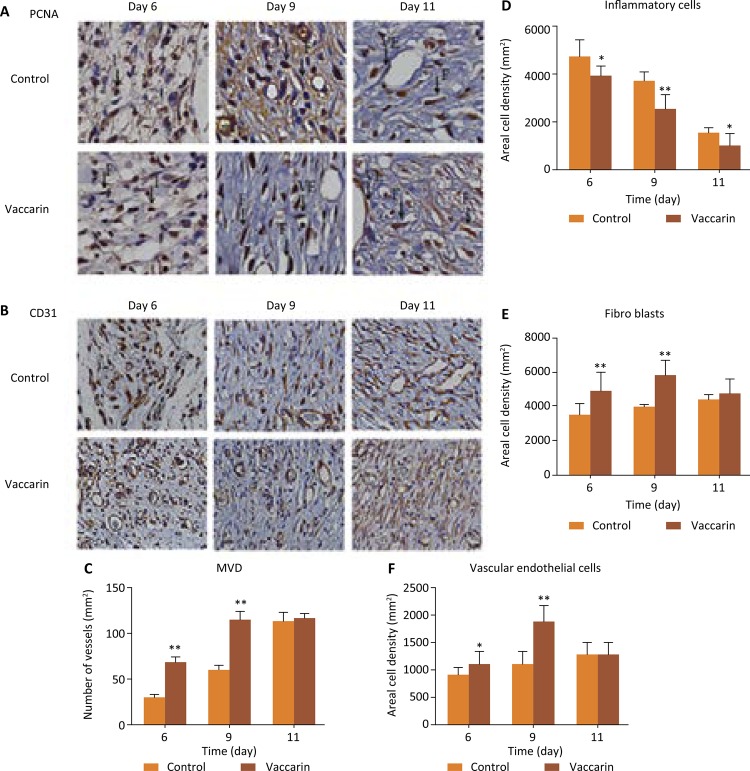
Vaccarin promoted angiogenesis. (A) Representative images of immunohistochemical PCNA staining of wound sections of control and vaccarin-treated rats on the 6th, 9th and 11th day after wounding (magnification x400). (B) Representative images of immunohistochemical CD31 staining of wound sections of control and vaccarin-treated rats on the 6th, 9th and 11th day after wounding (magnification x200). (C) Effect of vaccarin on the angiogenesis of rats was evaluated by MVD/CD31 compared with the blank control groups. (D-F) Representative of the inflammatory cells, fibroblasts and endothelial cell count from 10 randomly chosen high-power fields (magnification x400) from Immunohistochemistry stained cutaneous wound sections.

To assess the role of vaccarin in promoting cell proliferation, we stained PCNA by immunohistochemistry to observe cell proliferation. PCNA is known to be closely associated with S phase and DNA replication of the cell cycle^19^ . As shown in Figure 4 A, and D-F, inflammatory cells proliferated significantly on day 6th in both the vehicle control group and the varicarin treated group. In addition, vaccarin treated group fibroblasts exhibited markedly proliferation and endothelial cells also had few proliferation. On the 9th day, the vaccarin treated group showed more fibroblasts and endothelial cells proliferation, but proliferation of inflammatory cells had reduced. In vehicle control group fibroblasts and endothelial cells had few proliferation, but inflammation cells still showed considerable proliferation. On the 11th day, the vaccarin-treated group wound presented more fibroblast and endothelial cells proliferation, but there was almost no inflammatory cells proliferation, while in the vehicle control group fibroblasts and endothelial cells were still in proliferating. From these results, we hypothesize that vaccarin may hasten wound healing by promoting the proliferation of endothelial cells and fibroblasts.

### Effects of vaccarin on angiogenesis

We also assessed the effects of vaccarin in accelerating angiogenesis. The neovascularization activity was measured based upon the MVD via detecting expression of CD31^20^ . Compared with the controls, the MVD of the vaccarin treated group exhibited a significant increase. MVD is a marker to assess the level of angiogenesis and an increase of MVD in skin tissue suggested fast-growing blood vessels^14^ . As shown in Figure 4 B-C, immunohistochemical staining with antibodies against CD31 revealed that the MVD significantly increased in the vaccarin-treated group compared with controls. These observations indicated that vaccarin has effects on promoting angiogenesis.

### Effects of vaccarin for activation receptor associated with angiogenesis

**Figure 5:**
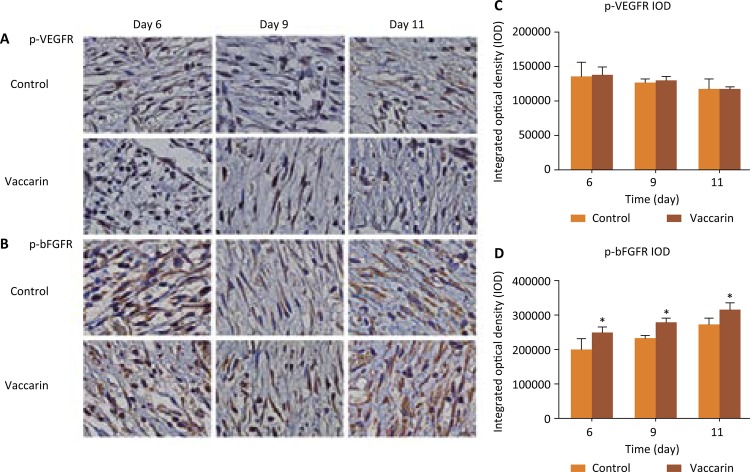
Vaccarin activated bFGFR signaling. (A and B) Representative images of immunohistochemical p-VEGFR and p-bFGFR staining of wound sections of control and vaccarin-treated rats on the 6th, 9th and 11th day after wounding (magnification x400). (C and D) Representative the statistic IOD of p-VEGFR and p-bFGFR respectively. Data are presented as the mean ± standard error of the mean from six independent experiments. * *P* <0.05 compared with the blank control group.

We also evaluated the effects of vaccarin for activation receptor associated with angiogenesis .Growth factors are biologically active polypeptides which are involved in cell growth differentiation, proliferation, migration and metabolism^21^ . From the Figure 5 B and D we can see that the expression of p-bFGFR had a significant difference in the vaccarin treated group as compared with control group ( *P* <0.05), while in Figure 5 A and C, there was no significant difference in the expressions of p-VEGFR. These results suggested that vaccarin may stimulate endothelial cell proliferation and promote angiogenesis through targeting the bFGF/bFGFR2 signaling pathway.

### Effects of vaccarin for activation proteins associated with angiogenesis

**Figure 6:**
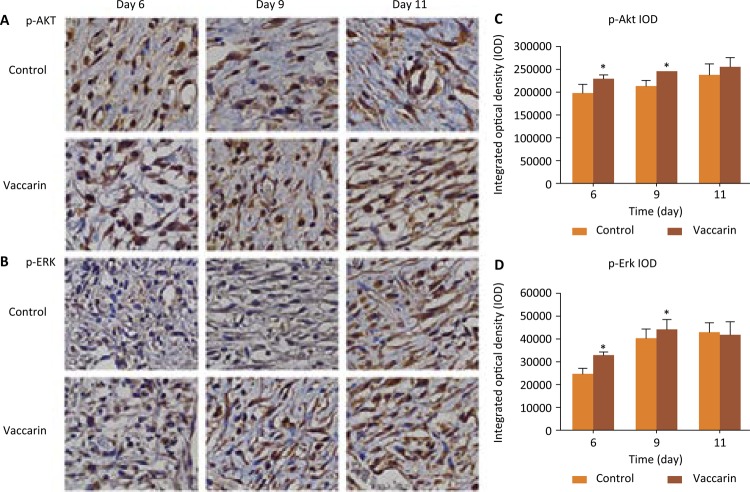
Vaccarin activated angiogenesis-associated channel proteins. (A and B) Representative images of immunohistochemical p-Akt and p-Erk staining of wound sections of control and vaccarin-treated rats on the 6th,9th and 11th day after wounding (magnification x400). (C and D) Compared with the vehicle control groupl p-Akt and p-Erk expression was higher following treatment with vaccarin. Data are presented as the mean ± standard error of the mean from six independent experiments.* *P* <0.05, ** *P* <0.01 compared with the vehicle control group.

To further explore the role of vaccarin in activating angiogenesis, we investigated related pathway proteins. The PI3K/AKT pathway is the center of many important cellular functions signal transmission, including cell adhesion, migration, and angiogenesis^22 - 24^ . Studies have shown that a variety of important growth factors through up-regulating the PI3K/AKT pathway plays its biological function in promoting angiogenesis^23^ . Akt protein is an important node protein in PI3K signaling pathways, which makes the PI3K/AKT/mTOR pathway activation by phosphorylation of Akt, then promoting angiogenesis. As shown in Figure 6 A and C, in the vaccarin-treated group the expressions of p-Akt were significantly higher than in the control group. These results suggested that PI3K/AKT pathway may be activated.

In the MAPK signaling pathways including 3 signaling pathways, the ERK signaling pathways are the most common. A recent study demonstrates that ERK-MAPK signaling is a key mediator of hyper activated FGFR2 signaling and may contribute to craniosynostosis^25^ . Hirudin regulates the expression of angiogenic and antiangiogenic factors via a cross-talk of p38 MAPK-ERK pathway to promote angiogenesis^26^ . As shown in Figure 6 B and D, in the vaccarin-treated group the expressions of p-Erk were markedly increased as compared with control group. These results suggested that the MAPK/ERK signaling pathways may be also activated. In addition, activation of Erk and Akt has been verified to correlate with angiogenesis^27^ . This further indicated that vaccarin was able to promote angiogenesis, by activating the MAPK/ERK and PI3K/AKT signaling *pathways.*

### Western blotting analysis

**Figure 7:**
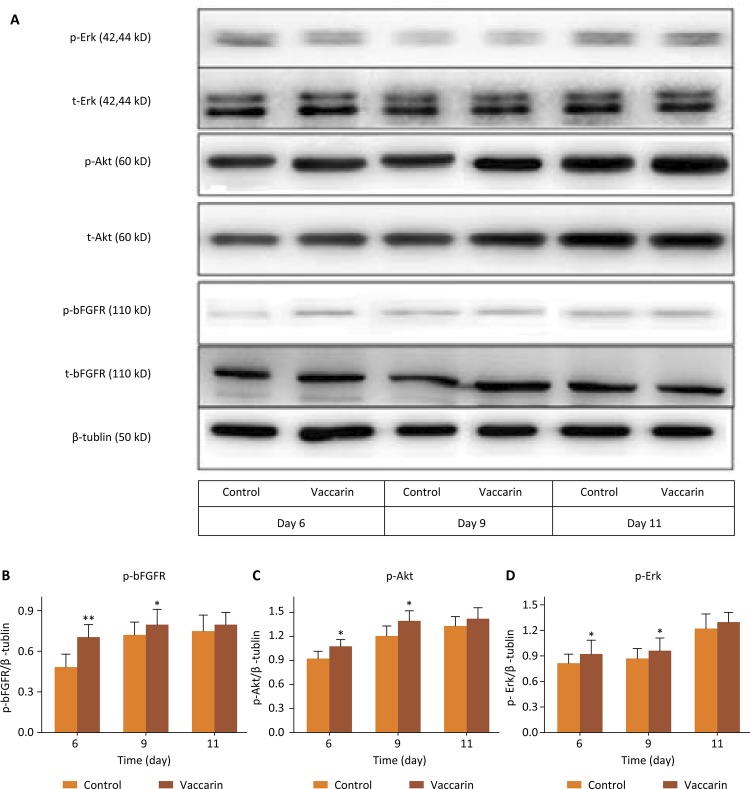
Effect of vaccarin on the expression of related proteins in skin of wound healing, compared with the vehicle control group* *P* <0.05, ** *P* <0.01.

In order to further elucidate the effects of vaccarin on promoting angiogenesis, we examined the key signaling molecules in the cell proliferation signaling pathway. Akt and Erk are closely related to angiogenesis, including migration, proliferation and survival of endothelial cells. As shown in Figure 7 (A-D), the Erk, Akt and bFGFR protein phosphorylation levels were significantly increased by vacccarin. These results revealed that vaccarin could promote phosphorylation of the key proteins in the cell proliferation, activating their signaling pathway and then affecting the phosphorylation of related target proteins in the downstream signaling pathway.

## Discussion

Wound healing is a worldwide issue, which causes heavy economic and healthcare burden^28^ . This issue has attracted the attention of an increasing number of researchers. In previous reports, traditional Chinese medicines such as curcumin, bromelain^29^ , *aloe vera* ^30^ , *aeschynomene indica L.* leaves^31^ , *verbascum speciosum* and astragaloside IV have been tried for the treatment of wounds. In addition, these drugs act on different stages of wound healing and achieve different healing effects. The results of this study showed that vaccarin could promote wound healing by inducing angiogenesis *in vivo* . The most interesting finding was that in the vaccarin treatment group, there was a large proliferation of fibroblasts during wound healing. Although Sun et al report that vaccarin can activate fgf-2-mediated FGFR1 signaling pathway in human microvascular endothelial cells^16^ , , we still cannot determine *in vivo* that vaccarin results in fibroblast proliferation, or that wound healing results in the proliferation of fibroblasts and endothelial.

Angiogenesis, the growth of new vasculature, may become promising therapeutic targets for wound healing^14^ . The proliferation, migration, and tube formation are coordinately involved in the formation of new blood vessels^32^ . In angiogenesis, the major angiogenic factors vascular endothelial growth factor (VEGF) and some minor ones, are involved in promotion and stabilization of new blood vessels^33^ . In the current study, we found that vaccarin exhibited the ability to promote angiogenesis at the wound site. We all know that CD31 and bFGFR coexist on vascular endothelial cells. Further study found that vascular endothelial cells significantly proliferated by fluorescent double staining of CD31 and bFGFR in the vaccarin-treatment group. These observations indicated that vaccarin has good effects on promoting angiogenesis.

Growth factors are biologically active polypeptides, which are involved in cell growth, differentiation, proliferation, migration and metabolism^21^ . As mentioned by previous research, bFGF is well known as a potent angiogenic factor that participates in the growth and migration of endothelial cells. It is released by mast cells, and stimulates the proliferation of endothelial cells and myocytes. bFGF plays an important role in angiogenesis and wound healing^34^ , and fibroblast growth factor receptor (FGFR)-mediating signaling is pivotal in angiogenesis and wound healing. The results of this study showed that bFGFR is activated by vaccarin in the wound site ( Fig. 5 ).The present findings seem to be consistent with other research that found that FGFR is activated in the process of wound healing^25^ . Intriguingly, published papers have established that foxc1 can promote the proliferation of fibroblast-like synoviocytes in rheumatoid arthritis via the PI3K/AKT signalling pathway^35^ .

The PI3K/AKT pathway is the center of many important cellular functions in signal transmission, including cell adhesion, migration, and angiogenesis^22^ . Studies have shown that a variety of important growth factors through the activation of the PI3K/AKT pathway plays a biological function in promoting angiogenesis^23^ . Akt protein is an important node protein in PI3K signaling pathways, which makes the PI3K/AKT/mTOR pathway activation by phosphorylation, then promoting angiogenesis^36^ . In the present study, our results demonstrated that vaccarin augmented the expression of p-Akt. We also found that the MAPK/ERK/extracellular signal was activated by vaccarin. Further studies are needed to elucidate the role of vaccarin in the whole phase of wound healing and to explore the relevant mechanism.

## Conclusions

Taken all results together, the present study is the first to demonstrate that vaccarin is able to induce angiogenesis and accelerate wound healing *in vivo* by increasing expressions of p-Akt, p-Erk and p-bFGFR. This process is mediated by MAPK/ERK and PI3K/AKT signaling pathways. Vaccarin has definitely shown to be a promising agent with great potential for wound healing.

## Footnotes

1 Research performed at Experimental Animal Center, Wuxi Medical College, Jiangnan University.
